# MsMTA regulates growth and metabolism in alfalfa (*Medicago sativa*) through m^6^A-mediated stabilization of metabolic transcripts

**DOI:** 10.1093/hr/uhag146

**Published:** 2026-04-16

**Authors:** Wenwu Qian, Mengzhuo Lin, Huayue Liu, Tingting Wang, JiaMin Cao, Gang Xu, Yunwei Zhang

**Affiliations:** College of Grassland Science and Technology, China Agricultural University, Beijing 100193, China; College of Grassland Science and Technology, China Agricultural University, Beijing 100193, China; College of Grassland Science and Technology, China Agricultural University, Beijing 100193, China; College of Grassland Science, Shanxi Agricultural University, Taigu 030801, China; College of Grassland Science and Technology, China Agricultural University, Beijing 100193, China; Institute of Leisure Agriculture, Shandong Academy of Agricultural Science, Jinan 250100, China; College of Grassland Science and Technology, China Agricultural University, Beijing 100193, China; Crop Research Institute of Xinjiang Uygur Autonomous Region Academy of Agricultural Sciences, Urumqi 830091, China; College of Grassland Science and Technology, China Agricultural University, Beijing 100193, China; College of Grassland Science and Technology, China Agricultural University, Beijing 100193, China

## Abstract

N6-methyladenosine (m^6^A) is a widespread RNA modification in eukaryotes, yet its contribution to growth and metabolic regulation in perennial leguminous forage crops remains poorly understood. Here, we identify MsMTA, a nuclear-localized m^6^A methyltransferase in alfalfa (*Medicago sativa*), as a key regulator of the global m^6^A landscape, plant architecture, and forage nutritional composition. Suppression of *MsMTA* expression reduced overall m^6^A levels and caused a pronounced dwarf phenotype. At the metabolic level, *MsMTA* knockdown lines showed a strong increase in soluble sugar content, accompanied by only a modest reduction in crude protein and decreased fiber, reflecting a reprogramming of carbon–nitrogen allocation that enhances agronomic quality in alfalfa. Integrated m^6^A sequencing and transcriptome profiling showed that m^6^A sites are preferentially enriched in the 3′UTR and that the expression of 549 genes is significantly altered; these genes are mainly involved in oxidation–reduction processes, small-molecule metabolic processes, and organonitrogen compound biosynthesis. Representative metabolic genes, such as *GS2* and *SDR1,* exhibited reduced m^6^A modification and decreased mRNA stability, directly linking MsMTA-dependent methylation to transcript turnover. Together, these findings establish MsMTA as a central node connecting the m^6^A epitranscriptome with carbon–nitrogen metabolic homeostasis in alfalfa, and indicate that targeted manipulation of MsMTA-dependent m^6^A methylation offers a potential route to develop alfalfa cultivars that maintain high protein content while substantially elevating soluble sugars, thereby improving forage yield and quality in leguminous crops.

## Introduction

Among RNA modifications, N6-methyladenosine (m^6^A) is the most abundant internal mark in eukaryotic messenger RNAs [1]. It contributes to post-transcriptional control of gene expression by affecting RNA stability [2, 3], splicing [4–6], nuclear export [4], and translational efficiency [7–9]. The deposition, removal, and recognition of m^6^A are mediated by three classes of proteins: writers that install the modification, erasers that remove it, and readers that bind m^6^A to modulate RNA fate [10, 11]. In mammals, METTL3 functions as the catalytic core of the m^6^A methyltransferase complex [12], whereas in plants, its homolog mRNA adenosine methyltransferase A (MTA) acts as the principal methyltransferase within a conserved complex comprising MTA, MTB, FIP37, VIR, and HAKAI*.* m^6^A methylation has been implicated in diverse physiological and developmental processes in plants. Components of the m^6^A methyltransferase complex (‘writers’) are required for embryogenesis [13, 14], leaf morphogenesis [15, 16], trichome development [16–18], root development [16, 19], fruit ripening [20, 21], and floral transition [15, 22–25].

m^6^A-dependent regulation also contributes to plant responses to abiotic stresses, including salinity [26, 27], drought [28, 29], and low temperature [30, 31]. Transcriptome-wide mapping in plants has shown that m^6^A sites are predominantly enriched within coding sequence (CDS) regions and 3′ untranslated regions (3′UTRs), a distribution pattern closely associated with the control of mRNA stability and translation [32, 33].

Despite extensive progress in model plants, the role of m^6^A methylation in perennial legume crops remains poorly understood. Alfalfa, an important perennial forage legume, is characterized by high biomass yield, excellent nutritional value, and nitrogen fixation ability [34, 35]. The stem elongation and lignification level of alfalfa directly determine its biomass productivity and forage quality. Excessive lignification decreases digestibility and protein content, whereas an unbalanced carbon–nitrogen allocation affects sugar and cell wall polysaccharide accumulation [36]. However, most studies on alfalfa development and nutrient metabolism have focused on hormonal or transcriptional regulation, while the epitranscriptomic mechanisms underlying these processes remain largely unexplored. In particular, the composition, localization, and regulatory function of m^6^A methyltransferase genes in alfalfa have not yet been systematically characterized.

In this study, we identify MsMTA as a putative m^6^A methyltransferase in *Medicago sativa*. Subcellular localization and sequence analyses show that MsMTA is confined to the nucleus and carries the conserved methyltransferase domain characteristic of the m^6^A writer complex. RNA interference (RNAi)–mediated knockdown of *MsMTA* suppressed stem elongation and altered nutrient allocation, leading to increased soluble sugar levels and reduced crude protein (CP) and acid detergent fiber (ADF) contents. Combined m^6^A-DRS and transcriptome profiling revealed that *MsMTA* silencing markedly reduced global m^6^A levels and shifted m^6^A enrichment toward the 3′UTR. Among genes with both differential methylation and expression, most followed a ‘Hypo–Down’ pattern (reduced m^6^A and decreased transcript abundance) and were enriched in pathways related to photosynthesis, redox homeostasis, small-molecule metabolism, and organonitrogen compound biosynthesis. Representative genes, including *GS2, LQY1, ATPC2*, and *SDR1*, showed concordant losses of m^6^A and mRNA accumulation, indicating that MsMTA-dependent m^6^A methylation supports photosynthetic electron transport and energy balance to sustain stem growth and metabolic homeostasis.

Collectively, our results demonstrate that MsMTA functions as a core component of the m^6^A writer complex in alfalfa and is essential for coordinating post-transcriptional regulation with metabolic homeostasis. To our knowledge, this is the first evidence in alfalfa that an m^6^A methyltransferase directly controls RNA methylation patterns to influence the stability of transcripts encoding key metabolic enzymes. We propose that the MsMTA–m^6^A module modulates 3′UTR methylation and mRNA stability to coordinate photosynthetic energy distribution, redox metabolism, and structural development. These findings not only establish MsMTA as a key regulator of stem growth and nutrient allocation in alfalfa but also provide insight into conserved epitranscriptomic mechanisms that couple growth and metabolism in perennial legumes.

## Results

### MsMTA is a nuclear-localized protein required for m^6^A methylation in *M. sativa*

**Figure 1:**
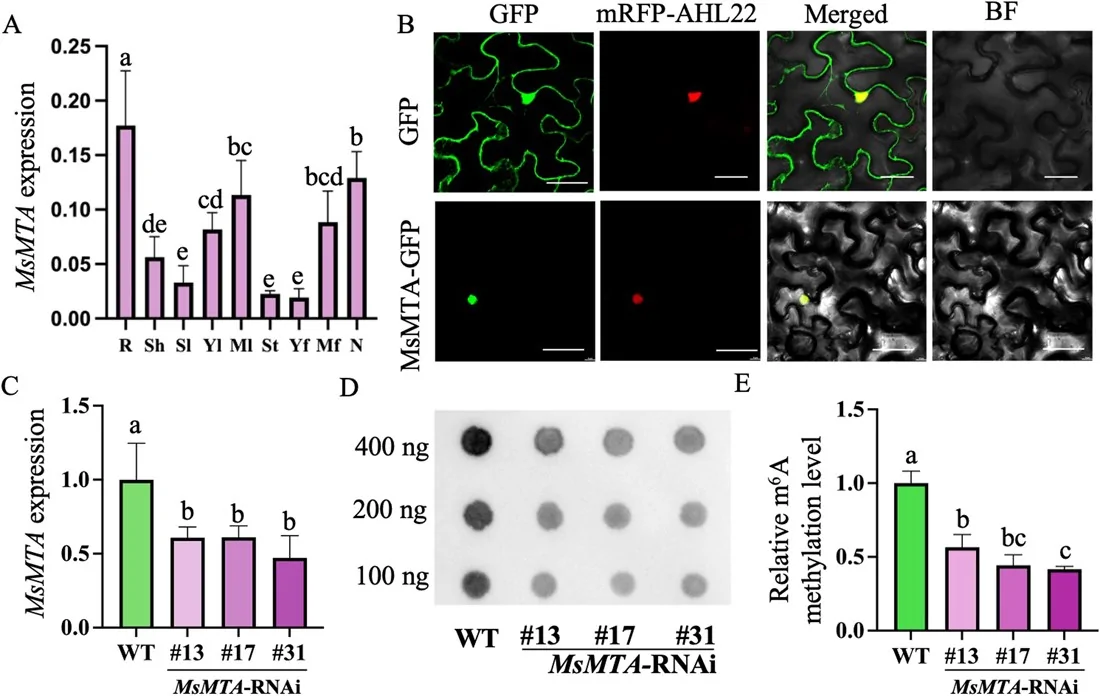
Functional analysis of MsMTA*.* (A) Expression patterns of *MsMTA* in various tissues of alfalfa. R, 10-day-old seedling roots; Sh, 10-day-old seedling shoots; Sl, 10-day-old seedling simple leaves; Yl, Young leaves (fold leaves); Ml, Mature leaves (opened leaves); St, stems; Yf, Young flowers; Mf, Mature flower; N, nodules. (B) Subcellular localization of MsMTA. 35S: GFP was used as a positive control. GFP: green fluorescent protein. Bars = 50 μm. (C) Relative expression levels of *MsMTA* in *MsMTA-*RNAi alfalfa lines. (D) Detection of total RNA m^6^A modification levels in *MsMTA-*RNAi alfalfa lines. (E) Quantification of m^6^A modifications from the dot blot in (D) using relative optical density measured by ImageJ.

MsMTA is the *M. sativa* homolog of MTA/METTL3 (Fig. S1A and C) and contains the conserved MT-A70 methyltransferase domain (Fig. S1B). RT-qPCR analysis detected *MsMTA* transcripts in all examined tissues, with relatively higher levels in roots, mature leaves, and nodules (Fig. 1A). In a transient expression assay in *Nicotiana benthamiana*, MsMTA–GFP fluorescence was predominantly observed in the nucleus (Fig. 1B).

To assess its biological function, RNAi and overexpression (OE) transgenic lines were generated via Agrobacterium-mediated transformation. Among four independent RNAi lines (Fig. S2A and S2B), three lines (*MsMTA*-RNAi#13, *MsMTA*-RNAi#17, and *MsMTA*-RNAi#31) with strong silencing efficiency were selected for phenotypic and physiological analysis. In these lines, *MsMTA* expression was reduced by 39.14%, 38.91%, and 52.84%, respectively, compared to wild-type (WT) plants (Fig. 1C). Similarly, among eight independent OE lines (Fig. S3A and B), three lines (*MsMTA*-OE#4, *MsMTA*-OE#21, and *MsMTA*-OE#23) with the highest expression levels were selected for phenotypic and physiological analysis. In these lines, *MsMTA* expression was 10.43-fold, 5.05-fold, and 7.47-fold higher, respectively, compared to WT plants (Fig. S3B).

RNA dot blot analysis further showed that global m_6_A abundance was significantly decreased in *MsMTA*-RNAi#13, #17, and #31 by 43.41%, 55.73%, and 58.35%, respectively, relative to WT (Fig. 1D and E). Collectively, these results indicate that MsMTA is required for efficient m6A deposition in *M. sativa*.

### MsMTA*,* a key m^6^A methyltransferase, regulates growth, development, and nutritional quality in *M. sativa*

**Figure 2:**
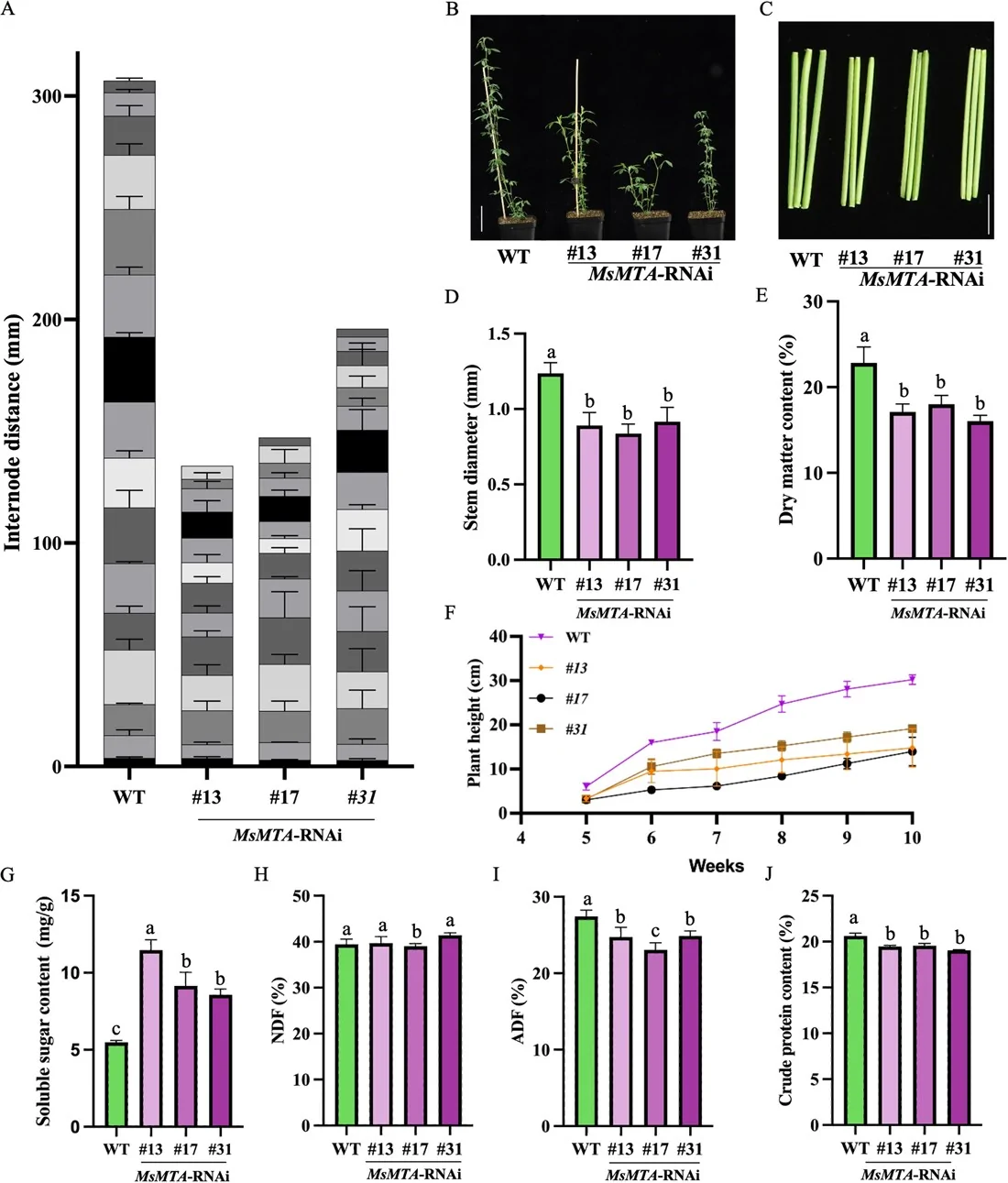
Silencing of *MsMTA* restricts stem growth and alters nutrient composition in alfalfa. (A) Comparison of internode length among 10-week-old WT plants and three independent *MsMTA*-RNAi lines (#13, #17, and #31). Error bars indicate the SD of at least five biological replicates. (B) Growth phenotype of WT and *MsMTA*-RNAi alfalfa plants. Scale bar = 5 cm. (C) Comparison of stem morphology between WT and *MsMTA*-RNAi lines. Scale bar = 5 mm. (D) Quantification of stem diameter in WT and *MsMTA*-RNAi plants. (E) Measurement of dry matter content in WT and *MsMTA*-RNAi plants. (F) Growth curves showing plant height over a 10-week period in WT and *MsMTA*-RNAi lines. (G) Determination of soluble sugar content in WT and *MsMTA*-RNAi lines. (H) Determination of NDF content in WT and *MsMTA*-RNAi lines. (I) Determination of ADF content in WT and *MsMTA*-RNAi lines. (J) Determination of CP content in WT and *MsMTA*-RNAi lines.

To investigate the role of MsMTA in *M. sativa* growth and development, we compared growth performance and nutritional traits between WT plants and three RNAi lines (*MsMTA*-RNAi#13, #17, and #31). All RNAi lines displayed a pronounced dwarf phenotype with significantly reduced plant height relative to WT (Fig. 2A, B, and  F). After 10 weeks of growth, internode length in WT plants was greater than in the RNAi lines (Fig. 2A), and stem diameter was also significantly higher in WT than in transgenic plants (Fig. 2C and D). In addition, the dry matter content of WT plants was substantially higher than that of the RNAi lines (Fig. 2E), whereas leaf size and leaf number did not differ significantly between WT and RNAi plants (Fig. 2B). In contrast, no significant differences were observed in plant height (Fig. S4B and F), stem diameter (Fig. S4C and S4E), internode length (Fig. S4A), and dry matter content (Fig. S4D) between the OE lines (*MsMTA*-OE#4, #21, and #23) and WT plants. These observations indicate that *MsMTA* silencing primarily restricts stem elongation and thickening, with little effect on leaf development.

Further analysis of nutritional quality traits (Fig. 2G–J) showed that soluble sugar content was significantly higher in all *MsMTA*-RNAi lines than in WT, with line #13 displaying the largest increase (Fig. 2G). In contrast, ADF content decreased by 9.30–15.89% in all RNAi lines, and CP content decreased by 5.11–7.55% relative to WT (Fig. 2I and J). Neutral detergent fiber (NDF) content was largely unchanged, apart from a slight reduction in line #17 (Fig. 2H). However, no significant differences were observed in the nutritional quality traits between the *MsMTA*-OE lines and WT plants (Fig. S5A–D).

Overall, *MsMTA* interference restricted stem growth and reshaped nutrient allocation. The RNAi lines exhibited a characteristic ‘high-sugar, modest low-protein, low-fiber’ nutritional profile, indicating that MsMTA contributes not only to stem growth regulation but also to carbon–nitrogen metabolism and cell wall component deposition. Although reduced ADF content in *MsMTA*-RNAi plants is consistent with partially improved forage quality, *MsMTA* silencing is likely to concomitantly decrease biomass production and stem mechanical strength.

### Effect of MsMTA on transcriptome-wide m^6^A methylation patterns in *M. sativa*

To investigate the impact of MsMTA on m^6^A methylation in *M. sativa*, we performed Oxford Nanopore Technologies direct RNA sequencing (ONT–DRS). Raw ONT reads were quality-filtered, retaining only those with a Phred quality score ≥10 and discarding lower-quality reads, thereby generating a high-confidence dataset for downstream analyses (Tables S1–S3).

**Figure 3:**
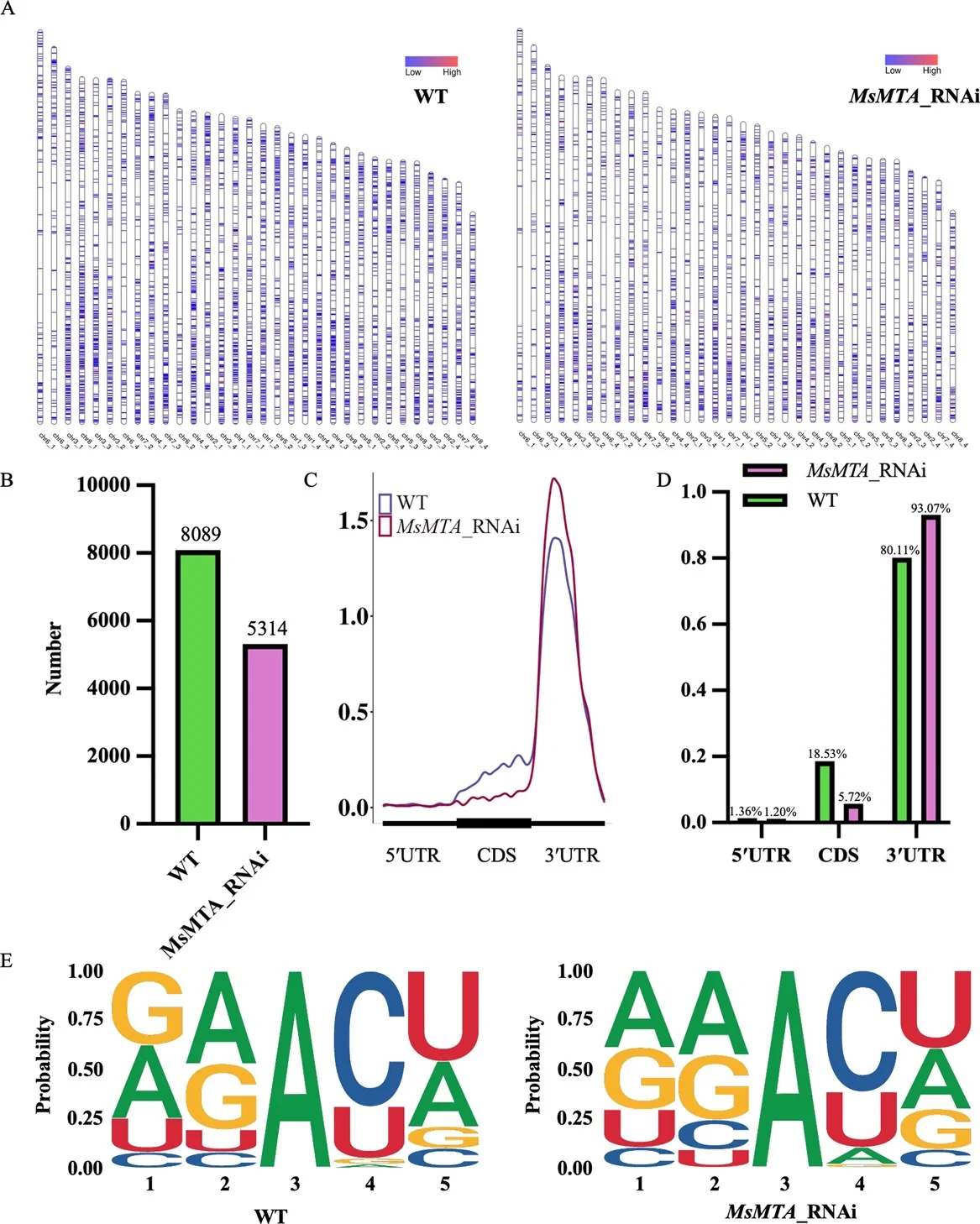
Distribution analysis of m^6^A modification sites in WT and *MsMTA*-RNAi lines. (A) Shows the distribution of m^6^A modification sites in WT and *MsMTA*-RNAi lines, with the color gradient representing the density of m^6^A modifications, where darker colors indicate higher-density modification sites and lighter colors indicate lower-density modification sites. (B) Bar graph comparing the number of m^6^A-modified sites in WT and *MsMTA*-RNAi lines. (C) Density plot illustrating the distribution of m^6^A modification sites in the 5′UTR, CDS, and 3′UTR regions in both WT and *MsMTA*-RNAi lines, highlighting the differences between the two. (D) Bar chart showing the proportional distribution of m^6^A peaks across different transcript regions (including CDS, 5′UTRs, and 3′UTRs), and around start and stop codons, comparing WT and *MsMTA*-RNAi. (E) Sequence logos representing the predominant m^6^A modification motifs in the WT and *MsMTA*-RNAi lines samples.

To explore the distribution pattern of m^6^A on chromosomes and the density distribution of m^6^A-modified transcripts, we generated a genome-wide map (Fig. 3A and Fig. S6). m^6^A-modified genes were detected on all 32 chromosomes, with a higher density of sites toward the chromosomal ends (Fig. 3A). Among these, chromosome Chr1_1 harbored the greatest number of m^6^A sites, whereas Chr8_4 contained the fewest. In addition, the total number of m^6^A-modified sites was markedly reduced in *MsMTA*–RNAi plants compared with WT plants (Fig. 3B).

Transcriptome-wide profiling revealed that m^6^A sites were predominantly enriched in 3′UTRs in both WT and *MsMTA*-RNAi plants (Fig. 3C). In WT plants, 18.53% of m^6^A sites were located in CDSs and 80.11% in 3′UTRs, whereas in *MsMTA*-RNAi plants these proportions shifted to 5.72% in CDSs and 93.07% in 3′UTRs (Fig. 3D).

Motif enrichment analysis identified the conserved sequence ‘GAACU’ as the predominant m^6^A recognition motif in both WT and *MsMTA*-RNAi plants (Fig. 3E). These results indicate that MsMTA is a key regulator of transcriptome-wide m^6^A modification patterns in *M. sativa*.

### MsMTA-mediated m^6^A regulation of gene expression and metabolic pathways in *M. sativa*

**Figure 4:**
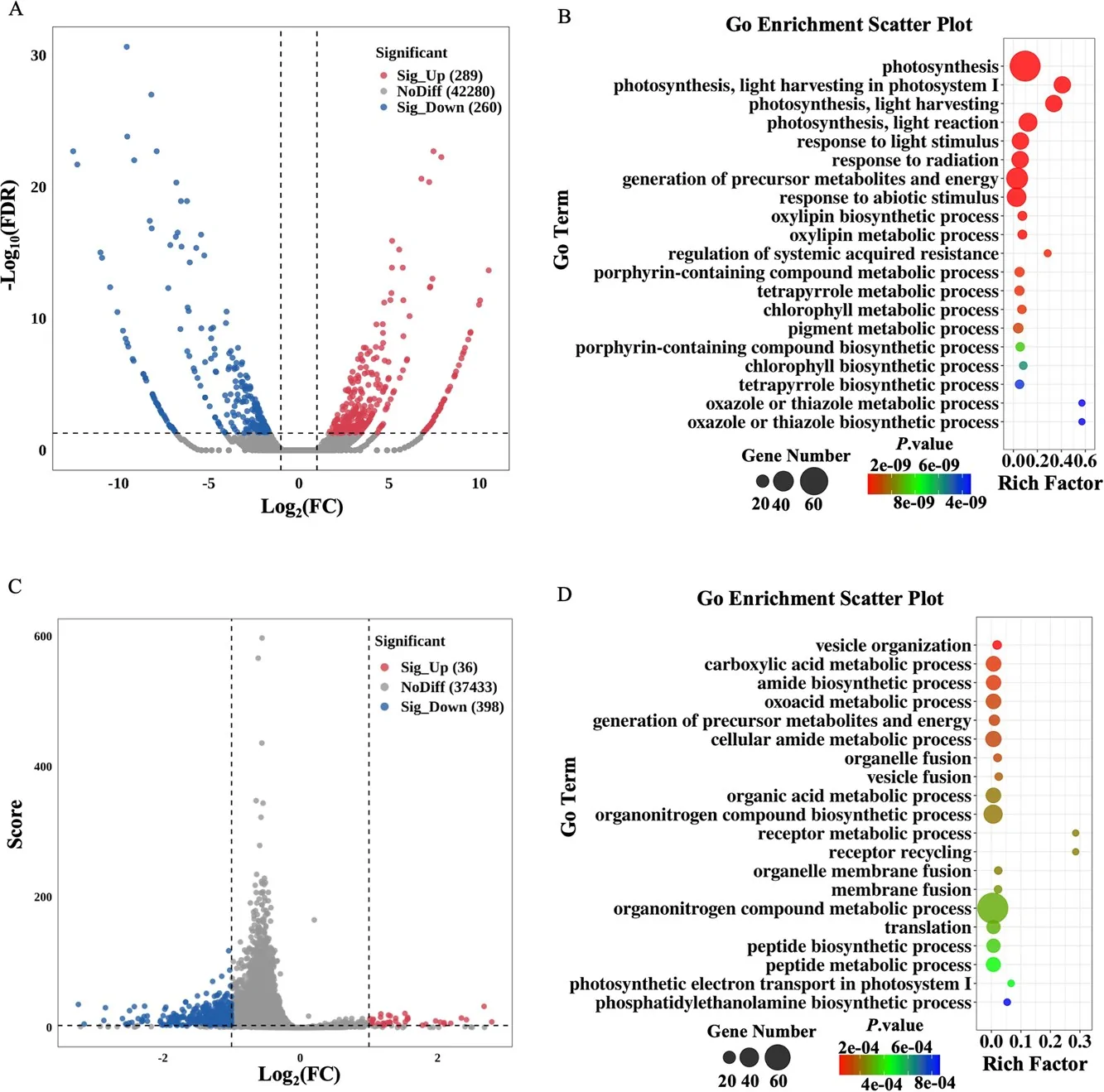
Differential gene expression and m6A modification in *M. sativa* under *MsMTA-*RNAi Conditions. (A) Volcano plot showing differential gene expression in *M. sativa* (WT vs. *MsMTA-*RNAi). Red points indicate significantly upregulated genes, and blue points indicate significantly downregulated genes. Significance was defined as an absolute |log₂(Fold Change)| > 1 and FDR < 0.05. (B) GO enrichment analysis of DEGs showing the top 20 enriched biological pathways. The gene ratio indicates the proportion of DEGs within each pathway relative to the total number of genes assigned to that pathway. (C) Volcano plot showing differential m^6^A modification in *M. sativa* (WT vs. *MsMTA-*RNAi). Red points represent significantly upregulated genes, and blue points denote significantly downregulated genes. Significance was defined as an absolute |log₂(Fold Change)| > 1 and a Score > 3. (D) GO enrichment analysis of differentially m6A-modified genes showing the top 20 enriched biological pathways. The gene ratio represents the proportion of differentially modified genes in each pathway relative to the total number of genes in that pathway.

To elucidate how MsMTA controls stem development and nutrient metabolism in *M. sativa*, we performed transcriptome sequencing on three independent *MsMTA*-RNAi lines (*MsMTA*-RNAi#13, #17, and #31). In line with the phenotypic and physiological analyses, suppression of *MsMTA* expression restricted stem elongation and altered nutrient allocation. RNA-seq identified 549 differentially expressed genes (DEGs), including 289 upregulated and 260 downregulated genes (Fig. 4A).

GO enrichment analysis of these DEGs (Fig. 4B) revealed significant overrepresentation of terms related to both biological processes and cellular components. Categories associated with photosynthesis and photosynthetic pigment metabolism were among the most highly enriched, suggesting that reduced *MsMTA* expression may impair photosynthetic capacity. DEGs were also enriched in pathways involved in cell wall biosynthesis, lignin metabolism, and hemicellulose biosynthesis, indicating that MsMTA affects metabolic networks underlying structural development and cell wall organization. Overall, these results indicate that MsMTA suppression alters stem development in *M. sativa* by modulating both photosynthetic activity and metabolic processes required for biomass formation.

To dissect the epitranscriptomic regulation associated with MsMTA, we analyzed genes with an m^6^A modification fold change >1 and a score >3. In total, 36 genes showed increased m^6^A methylation, whereas 398 genes displayed reduced methylation in *MsMTA*-RNAi plants (Fig. 4C). GO enrichment analysis of these differentially m^6^A-modified genes (Fig. 4D) revealed significant enrichment in processes related to vesicle organization and carboxylic acid metabolism, suggesting that MsMTA may contribute to growth and metabolic homeostasis through vesicular trafficking and organic acid metabolism. Genes involved in membrane fusion, organelle organization, and photosynthetic electron transport in photosystem I were also enriched, pointing to roles for MsMTA in energy metabolism, membrane dynamics, and photosynthetic regulation.

Genes involved in organelle fusion were strongly enriched, supporting a role for MsMTA in post-transcriptional control via m^6^A modification and in the regulation of genes linked to energy metabolism, cellular remodeling, and intracellular transport. Taken together, these data support a central role for MsMTA in coordinating stem development and nutrient allocation in *M. sativa* through combined transcriptional and epitranscriptomic regulation.

### MsMTA modulates oxidation–reduction, small molecule metabolic, and organonitrogen biosynthetic pathways through m^6^A-mediated regulation

**Figure 5:**
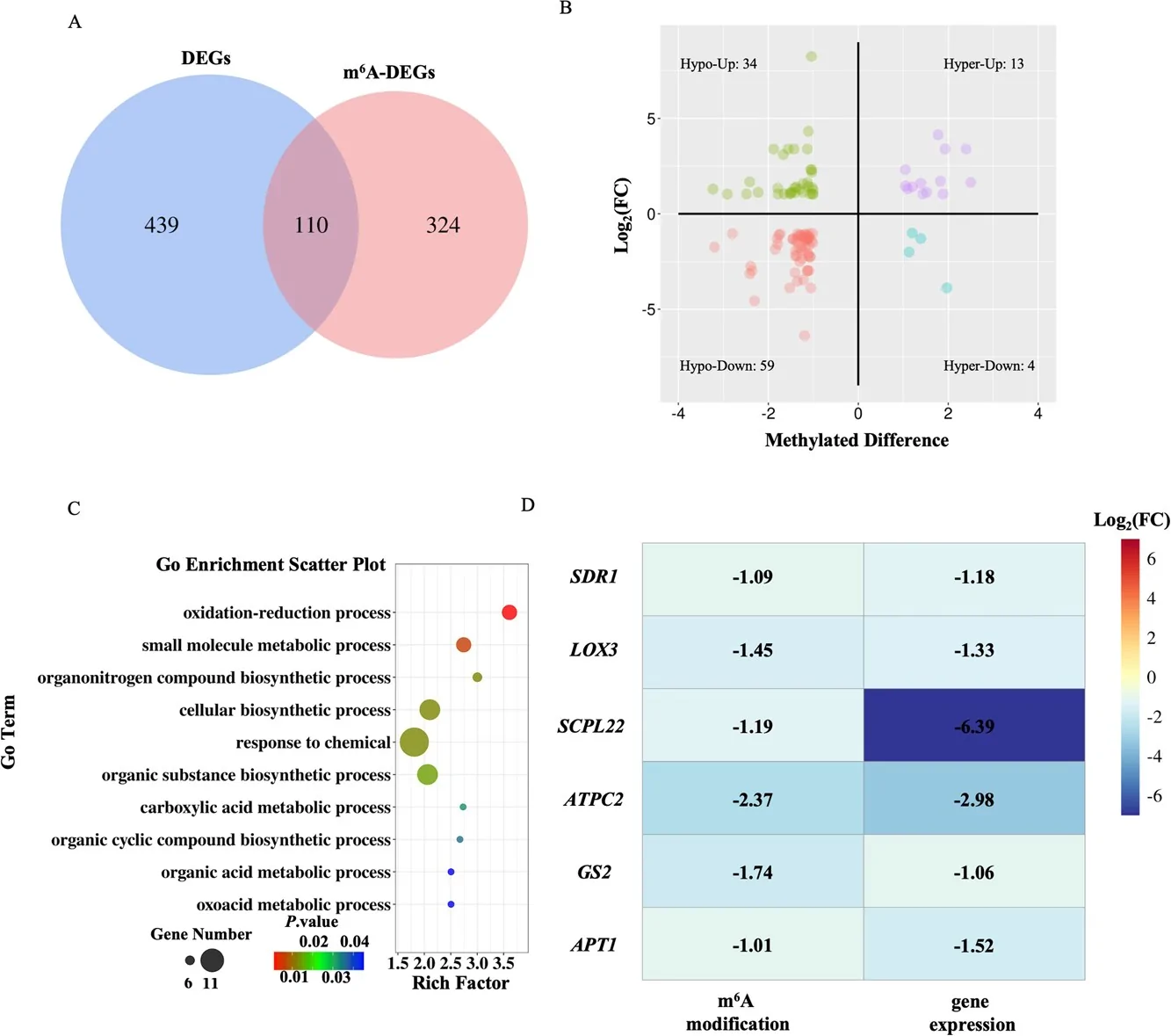
Association analysis between m^6^A modification and gene expression. (A) Overlap analysis between differentially methylated m^6^A-modified genes (m^6^A-DEGs) and DEGs. (B) Quadrant plot illustrating the correlation between m^6^A methylation changes and gene expression. Genes with increased methylation and upregulation are labeled ‘Hyper-up,’ those with increased methylation and downregulation ‘Hyper-down,’ genes with decreased methylation and upregulation ‘Hypo-up,’ and those with decreased methylation and downregulation ‘Hypo-down.’ (C) GO enrichment analysis of genes in the ‘Hypo-Down’ quadrant, highlighting the top 10 pathways enriched with hypomethylated and downregulated genes. (D) Heatmap showing the correlation between m^6^A methylation levels and transcript abundance of DEGs involved in the oxidation–reduction process, small molecule metabolic process, and organonitrogen compound biosynthetic process. Key representative genes are annotated, including Short-Chain Dehydrogenase/Reductase 1 (*SDR1*), Lipoxygenase 3 (*LOX3*), Serine Carboxypeptidase-Like 22 (*SCPL22*), ATP Synthase γ Subunit 2 (*ATPC2*), Glutamine Synthetase 2 (*GS2*), and Adenine Phosphoribosyltransferase 1 (*APT1*).

To determine how MsMTA affects transcriptomic and epitranscriptomic regulation, we compared the 549 DEGs with 434 genes carrying differential m^6^A modification sites and identified 110 overlapping genes that showed changes in both expression and m^6^A methylation (Fig. 5A). Integrated analysis of the m^6^A methylome and transcriptome categorized these genes into four groups based on concordant or discordant changes in methylation and expression: ‘Hyper–Up’ (increased methylation and expression), ‘Hypo–Up’ (decreased methylation and increased expression), ‘Hypo–Down’ (decreased methylation and expression) and ‘Hyper–Down’ (increased methylation and decreased expression) (Fig. 5B). Among these, the ‘Hypo–Down’ group contained 59 genes, accounting for ~54% of all methylated expressed genes and DEGs. The predominance of this class indicates that MsMTA suppression mainly causes downregulation of a subset of hypomethylated genes, which is likely to contribute to the growth inhibition observed in *MsMTA*-RNAi plants.

GO enrichment analysis of Hypo–Down genes showed significant enrichment in oxidation–reduction processes, small-molecule metabolic processes, and organonitrogen compound biosynthesis (Fig. 5C). These categories indicate that MsMTA contributes to the regulation of redox homeostasis and nitrogen-related metabolism.

Within these pathways, several representative genes exhibited concurrent loss of m^6^A methylation and transcriptional downregulation, including *GS2* (glutamine synthetase 2, involved in ammonium reassimilation), *SDR1* (short-chain dehydrogenase/reductase, contributing to NAD(P)H balance), *LQY1* (low quantum yield protein 1, required for PSII assembly and repair), *SCPL22* (serine carboxypeptidase-like 22, associated with small-molecule modification), *LOX3* (lipoxygenase 3, involved in lipid oxidation and jasmonate biosynthesis), *ATPC2* (ATP synthase γ subunit, mediating photophosphorylation) and *APT1* (adenine phosphoribosyltransferase 1, functioning in the purine salvage pathway) (Fig. 5D).

In summary, these genes are mainly involved in photosynthetic electron transport and redox regulation, indicating that MsMTA-dependent m^6^A modification is critical for maintaining photosynthetic efficiency and metabolic homeostasis. The concerted downregulation of redox- and energy-related pathways in the Hypo–Down group points to a global attenuation of cellular metabolism in *MsMTA*-RNAi plants, which is likely to contribute to the observed inhibition of stem growth.

### Validation of altered m^6^A enrichment, transcript levels, and mRNA stability of representative genes in *MsMTA*-RNAi lines

**Figure 6:**
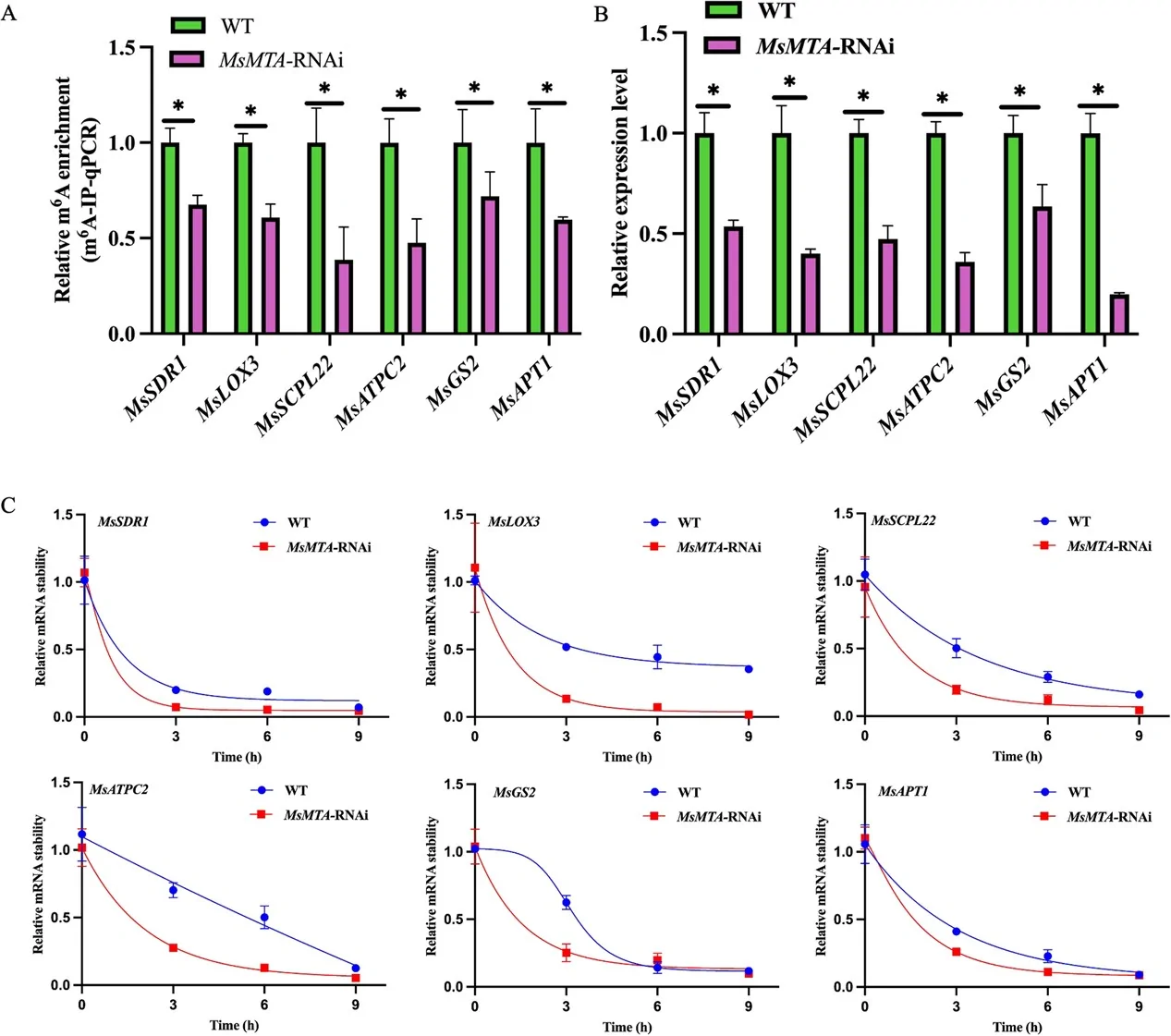
Validation of changes in m^6^A modification levels, transcript abundance, and mRNA stability of genes involved in three major metabolic pathways in *MsMTA*-RNAi alfalfa. (A) m^6^A enrichment levels of genes related to oxidation–reduction processes (*MsSDR1*, *MsLOX3*), small-molecule metabolic processes (*MsSCPL22*, *MsATPC2*), and organonitrogen compound biosynthetic processes (*MsGS2*, *MsAPT1*) were determined by m^6^A immunoprecipitation followed by quantitative PCR (m^6^A-IP-qPCR). (B) Relative expression levels of these genes were quantified by RT-qPCR using *MsACTIN* as the reference gene. (C) To evaluate mRNA stability, WT and *MsMTA*-RNAi plants were treated with 50 μg/ml actinomycin D, and RNA was extracted at different time points for RT-qPCR analysis. *MsACTIN* was used as the internal reference. Data are presented as the mean ± SEM of three biological replicates. Asterisks indicate significant differences (*P* < 0.05, Student's *t*-test).

To validate the sequencing results, m^6^A-IP–qPCR and RT-qPCR were used to assess m^6^A enrichment and transcript abundance for representative genes from three major metabolic pathways: oxidation–reduction processes (*MsSDR1*, *MsLOX3*), small-molecule metabolism (*MsSCPL22*, *MsATPC2*), and organonitrogen compound biosynthesis (*MsGS2*, *MsAPT1*). In agreement with the nanopore-based m^6^A-DRS data, all tested genes showed significantly reduced m^6^A enrichment together with significantly lower transcript levels in *MsMTA*-RNAi plants (Fig. 6A and B). Analysis of m^6^A enrichment levels and transcript abundance in the *MsMTA*-OE strain showed a significant increase in m^6^A modification for most genes, correlating with upregulation of transcript abundance in the majority of genes. However, a few genes exhibited no significant changes (Fig. S7A and B). These results indicate that loss of MsMTA-dependent m^6^A methylation compromises transcript accumulation across multiple metabolic pathways.

Given the close relationship between m^6^A modification and mRNA stability, actinomycin D assays were further conducted to assess transcript decay rates. Compared with the WT, *MsMTA*-RNAi plants showed accelerated degradation of *MsSDR1*, *MsLOX*3, *MsSCPL22*, *MsATPC2*, *MsGS2*, and *MsAPT1* transcripts (Fig. 6C). In contrast, in the *MsMTA*-OE strains, the degradation rates of these transcripts were partially slowed, while others showed no significant changes (Fig. S7C). These findings suggest that the observed alterations in these genes are closely associated with mRNA stability. Collectively, these findings demonstrate that MsMTA regulates gene expression by maintaining m^6^A-dependent mRNA stability and plays a pivotal role in the metabolic regulation of alfalfa.

## Discussion

*Medicago sativa* is an important perennial legume widely cultivated across the world [37]. With the advancement of high-throughput sequencing technologies, researchers can now efficiently identify key genes involved in plant growth and development. N^6^-methyladenosine (m^6^A) methylation is one of the most prevalent epitranscriptomic modifications, found across viruses and eukaryotes [38]. This modification regulates gene expression by influencing mRNA alternative splicing, selective translation, translational efficiency, and transcript stability, thereby affecting plant growth and development [20, 39, 40]. Oxford nanopore direct RNA sequencing (ONT-DRS) provides a powerful approach for profiling m^6^A modifications at the whole-transcriptome level, enabling accurate quantification of methylation across genes. Based on this platform, we conducted integrated m^6^A-DRS and RNA-seq analyses in *MsMTA*-RNAi and WT alfalfa plants to elucidate the regulatory mechanisms of m^6^A methylation in plant growth and metabolism. Our results revealed that m^6^A modification regulates alfalfa growth and nutritional quality by affecting the stability of transcripts associated with redox metabolism, small-molecule metabolism, and organonitrogen compound biosynthesis.

### MsMTA positively regulates stem growth and nutrient metabolism in alfalfa

In this study, we identified MsMTA as a core m^6^A methyltransferase in *M. sativa*. Silencing *MsMTA* caused a pronounced dwarf phenotype, including reduced plant height, shortened internodes, thinner stems, and decreased dry biomass accumulation (Fig. 2A–E), demonstrating that MsMTA-mediated m^6^A methylation is required for normal stem elongation and biomass formation. In line with this, the *MsMTA*-RNAi phenotype resembles MTA/METTL3 mutants reported in *Arabidopsis thaliana* [41], *Oryza sativa* [42], and *Sorghum* [43], supporting a conserved role of m^6^A writers in regulating cell elongation and organ development. Notably, although *MsMTA* OE increased global m^6^A levels, it did not result in obvious morphological changes, suggesting that elevating MsMTA alone is insufficient to enhance growth under our experimental conditions. This asymmetry is consistent with a buffered m^6^A homeostatic regime in which moderate increases can be accommodated by the regulatory network, whereas reductions more readily impair transcript stability and downstream regulatory programs, thereby producing stronger developmental defects.

Because internode elongation is typically governed by phytohormone signaling, we examined hormone-associated signatures in our transcriptome data. GO enrichment revealed only a limited hormone-related signal, with ‘ethylene metabolic process’ and ‘ethylene biosynthetic process’ enriched, whereas auxin-, gibberellin-, and brassinosteroid-related terms were not significantly enriched at the transcript level. This pattern does not exclude hormonal mediation, which may be stem-specific and/or occur via changes in hormone abundance or post-transcriptional regulation; however, it suggests that strong transcriptional activation of auxin/GA/BR pathways is not a dominant feature in leaf transcriptomes in our system. In parallel, integrated transcriptome and m^6^A profiling highlighted extensive alterations in redox-related processes, small-molecule metabolism, and organonitrogen compound biosynthesis, supporting the notion that MsMTA influences growth partly through metabolic remodeling. As metabolic pathways provide essential substrates and energy for cell division, cell expansion, and cell wall biosynthesis during stem development, disruption of carbon–nitrogen balance and energy homeostasis could impose a growth-limiting constraint on internode elongation and plant height. Consistently, *MsMTA*-silenced plants showed coordinated changes in forage quality, with increased soluble sugars but reduced CP and ADF contents (Fig. 2G–J), further supporting a role for MsMTA in coupling growth with nutrient metabolism in alfalfa.

### Conserved distribution pattern of m^6^A and its alteration in *MsMTA*-silenced plants

The m^6^A-DRS data revealed that m^6^A sites in alfalfa are mainly enriched around stop codons and within 3′UTRs (Fig. 3C and D), a pattern consistent with that observed in Arabidopsis, rice, maize, wheat, and tomato [44–47]. However, studies in strawberry, apple, and pak choi have shown that m^6^A modifications can also be highly enriched within CDS regions [20, 48, 49]. In alfalfa, *MsMTA* silencing caused a redistribution of m^6^A sites from CDS regions toward 3′UTRs (Fig. 3C), a shift typically associated with reduced transcript stability. This observation suggests that MsMTA enhances mRNA stability and translational efficiency by promoting the recruitment of m^6^A ‘reader’ proteins, such as ECT-like factors, to methylated transcripts.

### MsMTA-mediated m^6^A methylation affects gene expression and transcript stability

In mammals, m^6^A enrichment within 3′UTRs is generally correlated with reduced gene expression [34]. However, in plants, the regulatory effects of m^6^A can vary depending on developmental stages or stress conditions. For example, salt stress–induced changes in m^6^A levels enhance global transcript abundance in Arabidopsis [26], while during tomato fruit development, m^6^A methylation negatively correlates with gene expression during ripening but positively correlates during fruit expansion [21, 46]. Similarly, our results show that m^6^A methylation in alfalfa can exert both positive and negative regulatory effects on gene expression, primarily through modulation of mRNA stability (Fig. 6C). Comparative analysis of genes containing m^6^A sites across multiple transcript regions revealed a complex relationship between methylation position and transcript abundance, indicating that the regulatory role of m^6^A is context-dependent. The shift in methylation patterns upon *MsMTA* silencing reduces transcript stability of key genes, underscoring the central role of MsMTA in maintaining RNA fate and expression homeostasis.

### MsMTA maintains transcript stability across multiple metabolic pathways

Previous studies have shown that SDR deficiency weakens plant responsiveness to IBA, thereby affecting growth [47], while LOX participates in lipid peroxidation and oxylipin biosynthesis in Opium poppy, compounds that act as signaling molecules promoting growth and development [48]. Our findings further demonstrate that MsSDR1 and MsLOX3, two genes associated with redox processes, exhibit significantly decreased m^6^A methylation and transcript abundance in *MsMTA*-RNAi plants (Fig. 5B–D), suggesting that they function in oxidative–reductive signaling critical for alfalfa development. Moreover, small molecules have been reported to interact with proteins and modulate plant metabolic processes [49]. Among nitrogen-related genes, GS2 (glutamine synthetase 2) is involved in nitrogen assimilation and amino acid biosynthesis, directly influencing plant growth phenotype [50], while APT1 contributes to purine nucleotide recycling and energy homeostasis [51]. In *MsMTA*-silenced plants, the reduced m^6^A levels on *MsSCPL22*, *MsATPC2*, *MsGS2*, and *MsAPT1* transcripts led to accelerated mRNA decay and downregulated expression, resulting in disrupted redox balance, nitrogen assimilation, and photosynthetic metabolism (Fig. 5B–D). Collectively, these findings establish MsMTA as a key regulator linking m^6^A methylation to metabolic pathway coordination and energy stability in alfalfa.

### Prospects for optimizing alfalfa biomass and forage quality through m^6^A methylation regulation

Although silencing *MsMTA* improved certain forage quality traits, it also resulted in reduced plant height and biomass, which may limit its direct application in alfalfa breeding. Future strategies may therefore focus on more precise manipulation of m^6^A regulatory pathways to achieve a better balance between biomass accumulation and forage nutritional quality. For example, tissue-specific or developmental stage-specific modulation of MsMTA activity may help improve forage quality while minimizing negative effects on plant growth. In addition, targeted manipulation of downstream m^6^A-regulated genes involved in carbon–nitrogen metabolism or cell wall biosynthesis may provide alternative approaches for simultaneously improving biomass production and forage quality in alfalfa. These findings suggest that fine-tuning m^6^A methylation may represent a promising strategy for improving complex agronomic traits in forage crops.

**Figure 7:**
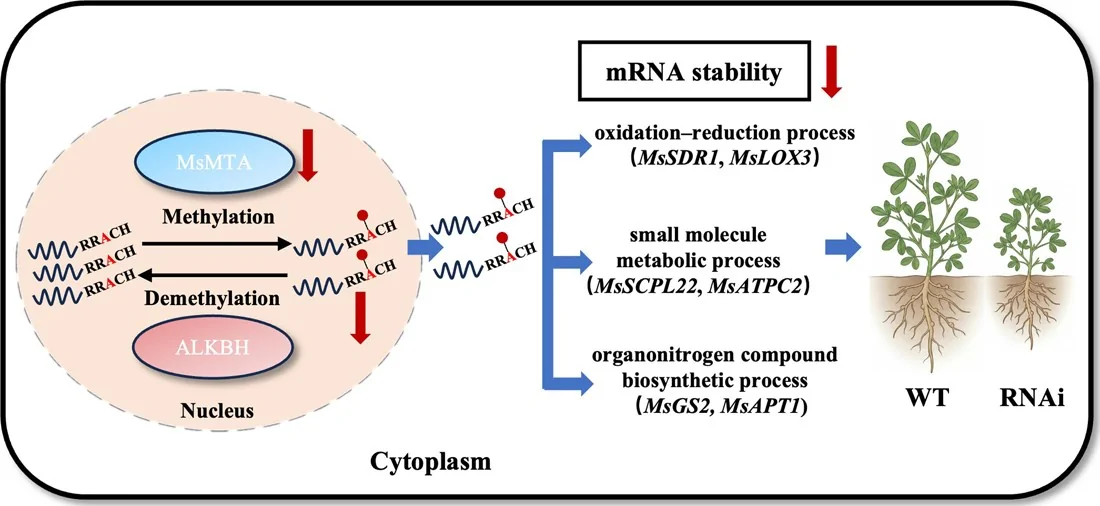
Proposed model of MsMTA-mediated m^6^A regulation in alfalfa. MsMTA functions as an m^6^A methyltransferase that deposits m^6^A marks on mRNAs involved in redox metabolism (*MsSDR1*, *MsLOX3*), small-molecule metabolism (*MsSCPL22*, *MsATPC2*), and organonitrogen biosynthesis (*MsGS2*, *MsAPT1*). ALKBH, an m^6^A demethylase, removes m^6^A marks from these mRNAs, regulating their stability and associated metabolic pathways. Silencing of *MsMTA* decreases overall m^6^A methylation levels, leading to reduced mRNA stability of these genes and the downregulation of associated metabolic pathways. Consequently, MsMTA deficiency disrupts energy metabolism and carbon–nitrogen balance, resulting in shortened internodes and reduced plant height in *M. sativa*.

Taken together, our study establishes MsMTA as a pivotal m^6^A methyltransferase in *M. sativa* that maintains transcript stability across multiple metabolic pathways. (Fig. 7) MsMTA-mediated m^6^A methylation positively regulates the stability and expression of transcripts associated with redox metabolism (*MsSDR1*, *MsLOX3*), small-molecule metabolism (*MsSCPL22*, *MsATPC2*), and organonitrogen compound biosynthesis (*MsGS2*, *MsAPT1*). Silencing *MsMTA* reduces global m^6^A levels, leading to transcript destabilization, downregulated metabolic activity, and impaired photosynthetic energy distribution, redox balance, and nitrogen assimilation. These alterations collectively disrupt carbon–nitrogen homeostasis and restrict stem elongation, resulting in shortened internodes and reduced biomass accumulation. Therefore, MsMTA functions as a key regulator linking m^6^A methylation to metabolic coordination and structural development in alfalfa, providing a molecular basis for improving forage yield and nutritional quality through post-transcriptional regulation.

## Materials and methods

### Plant materials and growth conditions

In this study, ‘Zhongmu No.1’ alfalfa cultivar was used as the plant material. Plants were grown in growth chambers or greenhouses under controlled environmental conditions with a constant temperature of 25°C, a 16-h light/8-h dark photoperiod, and 60% to 70% relative humidity. The plants were cultivated in plastic pots (7 cm in length) filled with vermiculite as the growth medium. Irrigation was provided with 1/4 strength Hoagland nutrient solution (pH 5.8–6.0). Plants were watered every 3–4 days to maintain adequate moisture in the vermiculite.

### Phenotypic measurements

WT and transgenic lines were propagated by stem cuttings and grown under identical conditions. Plant height was recorded weekly from Week 5 to Week 10 after transplantation and was defined as the distance from the soil surface to the apex of the main shoot. Measurements were initiated at Week 5 because alfalfa plants had established stable vegetative growth by this stage, allowing reliable assessment of stem elongation and plant architecture. Internode length was measured on the main stem as the distance between two adjacent nodes. Stem diameter was measured using a digital caliper at a fixed position (the midpoint of the fourth internode above the soil surface). For each line, five biological replicates were used.

### Vector construction and alfalfa transformation

The full-length CDS of *MsMTA* was amplified from ‘Zhongmu No.1’ alfalfa. For OE analysis, the *MsMTA* CDS was cloned into the binary vector pMDC32, driven by the CaMV 35S promoter.

For RNAi, a 200-bp fragment specific to the *MsMTA* CDS was amplified and inserted into the pK7GWIWG2D(II) vector using attL/attR Gateway recombination (Invitrogen). All primer sequences used in this study are listed in Table S4.

Recombinant plasmids were introduced into *Agrobacterium tumefaciens* strain EHA105 via the freeze–thaw method and subsequently used for *Agrobacterium*-mediated transformation of ‘Zhongmu No.1’ alfalfa leaf explants to generate transgenic plants.

### Subcellular localization

To determine the subcellular localization of *MsMTA*, the CDS without the stop codon was fused in-frame to green fluorescent protein (GFP) at the C-terminus and cloned into the pMDC83 vector. The resulting construct (35S:MsMTA-GFP) and the GFP control (35S:GFP) were transformed into *Agrobacterium tumefaciens* strain GV2260.

*Agrobacterium* cultures harboring each construct were infiltrated into leaves of 4-week-old *Nicotiana benthamiana* plants. GFP fluorescence signals were observed 48–60 h after infiltration using a confocal laser scanning microscope (Nikon A1, Japan).

### Polyadenylated RNA preparation

Total RNA was extracted from leaf tissues frozen in liquid nitrogen using TRIzol reagent (Invitrogen, CA, USA). RNA concentration and purity were measured with a NanoDrop ND-1000 spectrophotometer (NanoDrop Technologies, Wilmington, DE, USA), and RNA integrity was assessed using an Agilent 2100 Bioanalyzer (Agilent Technologies, CA, USA). Samples with concentrations above 50 ng/μl, RNA integrity numbers (RIN) greater than 7.0, OD260/280 ratios above 1.8, and total yields exceeding 50 μg were used for subsequent analyses. Poly(A) + RNA was isolated using Dynabeads Oligo(dT) following the manufacturer's protocol (Thermo Fisher Scientific, USA; Cat. No. 25-61 005).

### Dot blot assay

Dot blot analysis was performed as previously described, with minor modifications [52]. Total RNA was isolated using TRIzol reagent (Invitrogen, CA, USA), denatured at 95°C for 5 min, and immediately chilled on ice for 2 min. RNA concentrations were determined using a NanoDrop 2000 spectrophotometer (Thermo Fisher Scientific). Equal amounts of RNA were spotted onto nitrocellulose membranes and cross-linked three times using an ultraviolet crosslinker (Scientz03-II, Ningbo Scientz Biotechnology). Membranes were blocked with 5% nonfat milk and incubated with an anti-m^6^A antibody (Synaptic Systems, #202003). Signal intensities were quantified using ImageJ software.

### Oxford Nanopore direct RNA sequencing (ONT-DRS)

For ONT-DRS analysis, total RNA from WT and *MsMTA*-RNAi plants (three biological replicates per group) was pooled for each condition. Sequencing libraries were prepared using the Direct RNA Sequencing Kit (SQK-RNA004, Oxford Nanopore Technologies, UK) according to the manufacturer's instructions. Sequencing was performed on MinION devices equipped with FLO-MIN004RA flow cells (Oxford Nanopore Technologies, UK). Two sequencing runs were conducted per condition. Basecalling was performed using Guppy software (v4.4.2) to meet DENA requirements. For each condition, at least 2.6 million reads with a minimum quality score of 8 were obtained. Sequencing runs were carried out for 48–54 h using flow cells containing approximately 1200 to 1500 active pores.

### DMR model and scoring details

In this study, the methylation differences between conditions were assessed using a likelihood ratio scoring method. For each region, methylation states were modeled using either a binomial distribution or a multinomial distribution. The score was calculated using the log-marginal likelihood ratio:


$$ \mathrm{Score}=\log \left(\frac{l{\theta}_a\left({X}_a\right)l{\theta}_a\left({X}_b\right)}{l{\theta}_{a+b}\left({X}_{a+b}\right)}\right) $$

where $l{\theta}_a\left({X}_a\right)$ and $l{\theta}_a\left({X}_b\right)$ are the marginal likelihoods under separate modeling for conditions a and b, and $l{\theta}_{a+b}\left({X}_{a+b}\right)$ is the marginal likelihood when both conditions are modeled together. The function *lθ (X)* represents the log marginal likelihood of the counts under the parameters *θ*, which are derived from a Beta distribution for the binary case or a Dirichlet distribution for the multinomial case.

The threshold of ‘Score > 3’ was used to identify regions with significant methylation differences between conditions. A score greater than 3 indicating a significant methylation change. The threshold was selected based on prior studies and is commonly used to detect robust methylation differences.

### m^6^A-IP-qPCR

m^6^A-IP-qPCR was conducted following previously published protocols [53]. Briefly, 50 μg of total RNA was fragmented to 200–300 nt using RNA Fragmentation Reagents (Invitrogen, AM8740). After fragmentation, RNA was purified by ethanol precipitation, and 10% was retained as input. The remaining RNA was incubated with 5 μg of anti-m^6^A antibody (Synaptic Systems, #202003) in immunoprecipitation buffer supplemented with high salt and RNase inhibitor for 2 h at 4°C. m^6^A-enriched RNA was captured using Dynabeads Protein A, eluted with m^6^A nucleoside buffer, and recovered by ethanol precipitation. Both IP and input RNA samples were reverse-transcribed using the HiScript III First Strand cDNA Synthesis Kit (Vazyme Biotech, R312–01, China). Quantitative PCR was performed on a qTower3G 96 system (Analytik Jena, Germany) using Taq Pro Universal SYBR qPCR Master Mix (Vazyme Biotech, Q712-02, China). Relative m^6^A enrichment was calculated using the 2^−ΔCt^ method and normalized to the input.

### RT-qPCR

Total RNA was extracted using the Beijing Huayueyang RNA Kit, and 1 μg of RNA was reverse-transcribed into cDNA using the PrimeScript RT Reagent Kit (Takara, RR047A) according to the manufacturer’s instructions. RT-qPCR was performed on a qTOWER^3^G system (Analytik Jena, Germany) with SYBR Green Supermix (Takara, RR420A). Relative gene expression levels were calculated using the 2^−ΔΔCt^ method [54]. *MsActin* (MsG0380016789.01.T01) was used as the internal reference gene.

### mRNA stability assay

mRNA stability assays were carried out as previously reported with minor modifications [28]. Leaves from WT and *MsMTA*-RNAi plants were harvested and treated with 50 μg/ml actinomycin D. Samples collected immediately after treatment were designated as the 0-h time point, while additional samples were collected at 3, 6, and 9 h. Total RNA was isolated as described above, and transcript abundance was determined by RT-qPCR using gene-specific primers.

### Statistical analyses

Statistical analyses were performed using SPSS Statistics 17.0 software. Data are presented as mean ± standard deviation (SD). Differences among groups were evaluated using one-way analysis of variance (ANOVA), followed by Duncan's multiple range test. Differences were considered statistically significant at *P* < 0.05, and significance levels are indicated by different letters.

## Supplementary Material

Web_Material_uhag146

## Data Availability

The data supporting the findings of this study are available within the article and its Supplementary Information files.
