# Supplementary material for: MsMTA regulates growth and metabolism in alfalfa (Medicago sativa) through m6A-mediated stabilization of metabolic transcripts
Source: Hortic Res. 2026 Apr 16;13(9):uhag146. doi: 10.1093/hr/uhag146 (PMC13503968; doi:10.1093/hr/uhag146)
Supplement: Supplementary file 1 [file web_material_uhag146.zip › Supplementary Materials.docx]

Supplementary Materials for

MsMTA regulates growth and carbon–nitrogen metabolism in alfalfa (*Medicago sativa*) through m^6^A-dependent stabilization of metabolic transcripts

WenWu Qian et al.

*Corresponding author. Email: xugang@cau.edu.cn; & zywei@cau.edu.cn

Figure S1. Evolutionary and structural conservation of MsMTA among plant MTA methyltransferases. (A) Phylogenetic tree of MsMTA and MTA proteins from several representative plant species, with MsMTA highlighted in red. (B) Schematic domain organization of MsMTA and its homologs. Colored boxes represent conserved domains, which are labeled in the key on the right, and *MsMTA* is indicated by the red dashed box.

(C) Multiple sequence alignment of MsMTA with Arabidopsis AtMTA and Brassica BrMTA proteins. Highly conserved amino acid residues are shaded in red, showing the strong conservation of characteristic MT-A70-type m^6^A methyltransferase motifs.

**Figure S2. Identification of the *MsMTA*-RNAi transgenic alfalfa.** (A): PCR detection of *MsMTA* gene in *MsMTA*-RNAi transgenic alfalfa. +: constructed vector as a positive control. WT: wild type. -: H_2_O was used as a template for a negative control. (B): The expression of *MsMTA* in *MsMTA*-RNAi transgenic alfalfa. *MsActin* was used as an internal standard. The WT was normalized to 1. Data are the means ± SD (n=3). Asterisks represent statistically significant differences as indicated by Student’s t-test, *P < 0.05.

Figure S3. Identification of the *MsMTA*-OE transgenic alfalfa. (A): PCR detection of *MsMTA* gene in *MsMTA*-OE transgenic alfalfa. +: constructed vector as a positive control. WT: wild type. -: H_2_O was used as a template for a negative control. (B): The expression of *MsMTA* in *MsMTA*-OE transgenic alfalfa. *MsActin* was used as an internal standard. The WT was normalized to 1. Data are the means ± SD (n=3). Asterisks represent statistically significant differences as indicated by Student’s t-test, *P < 0.05.

**Figure S4. Overexpression of *MsMTA* does not significantly affect stem growth traits in alfalfa.** (A) Comparison of internode length among 10-week-old wild-type (WT) plants and three independent *MsMTA*-OE lines (#4, #21, and #23). Error bars indicate the standard deviation (SD) of at least five biological replicates. (B) Growth phenotype of WT and *MsMTA*-OE alfalfa plants. Scale bar = 5 cm. (C) Comparison of stem morphology between WT and MsMTA-OE lines. Scale bar = 5 mm. (D) Quantification of stem diameter in WT and *MsMTA*-OE plants. (E) Measurement of dry matter content in WT and *MsMTA*-OE plants. (F) Growth curves showing plant height over a 10-week period in WT and *MsMTA*-OE lines.

**Figure S5. Determination of nutritional indices of alfalfa.** (A) Determination of soluble sugar content in WT and *MsMTA*-OE lines. (B) Determination of neutral detergent fiber (NDF) content in WT and *MsMTA*-OE lines. (C) Determination of acid detergent fiber (ADF) content in WT and *MsMTA*-OE lines. (D) Determination of crude protein (CP) content in WT and *MsMTA*-OE lines.

**Figure S6. Transcript density distribution plot.** From outside to inside, they represent the chromosome, all transcripts, known transcripts, and novel transcripts.

**Figure S7. Validation of changes in m⁶A modification levels, transcript abundance, and mRNA stability of genes involved in three major metabolic pathways in *MsMTA*-OE alfalfa.** (A) m^6^A enrichment levels of genes related to oxidation–reduction processes (*MsSDR1,* *MsLOX3*), small-molecule metabolic processes (*MsSCPL22*, *MsATPC2*), and organonitrogen compound biosynthetic processes (*MsGS2*, *MsAPT1*) were determined by m^6^A immunoprecipitation followed by quantitative PCR (m^6^A-IP-qPCR). (B) Relative expression levels of these genes were quantified by RT-qPCR using *MsACTIN* as the reference gene. (C) To evaluate mRNA stability, wild-type and *MsMTA*-OE plants were treated with 50 μg/mL actinomycin D, and RNA was extracted at different time points for RT-qPCR analysis. *MsACTIN* was used as the internal reference. Data are presented as the mean ± SEM of three biological replicates. Asterisks indicate significant differences (P < 0.05, Student’s t-test).

**Table S1. Statistics of sequencing data volume information.**

| Sample | Type | TotalBase | TotalReads | MaxLen | AvgLen | N50 | L50 | N90 | L90 | meanQ |
| --- | --- | --- | --- | --- | --- | --- | --- | --- | --- | --- |
| WT | all | 1,160,711,789 | 1,201,564 | 261,280 | 966 | 1,053 | 367,091 | 627 | 922,546 | 16.02 |
| WT | pass | 1,119,923,421 | 1,141,392 | 261,280 | 981.19 | 1,058 | 354,082 | 638 | 885,683 | 16.52 |
| RNAi | all | 689,897,989 | 693,558 | 298,359 | 994.72 | 1,201 | 193,456 | 615 | 512,593 | 17.5 |
| RNAi | pass | 667,035,558 | 660,146 | 298,359 | 1,010.43 | 1,206 | 187,235 | 624 | 493,726 | 18.08 |

**Table S2. Statistics of read mapping results.**

| Sample | Total reads | mapped reads | map_rate |
| --- | --- | --- | --- |
| WT | 1,141,392 | 1,101,381 | 96.49% |
| RNAi | 660,146 | 635,396 | 96.25% |

**Table S3. Statistics of consensus sequence information.**

| Sample Name | Seq Num | Mean Length(bp) | N50(bp) | Max Length(bp) |
| --- | --- | --- | --- | --- |
| WT | 11,831 | 1,141.00 | 1,533 | 7,117 |
| RNAi | 9,268 | 1,122.50 | 1,533 | 6,937 |

**Table S4. List of primer sequences.**

| Name | primer sequence (5′-3′) | Usage |
| --- | --- | --- |
| *qMsMTA-F* | CTTCAGCGAATAATTAGAACTGG | RT-qPCR |
| *qMsMTA-R* | TTCAGCGACAATAACATCGG |  |
| *qMsAction-F* | CAAAAGATGGCAGATGCTGAGGAT | Reference |
| *qMsAction-R* | CATGACACCAGTATGACGAGGTCG |  |
| *pMDC83-MTA-F* | acctcgactctagaaATGGACATGGAGACAGATGAAG | Subcellular localization |
| *pMDC83-MTA-R* | ccgggccccccctcgaGTTAGTGTCCACGTCAATGACC |  |
| *MsMTA-F* | AACTCCGTCGTATCCGCTTCC | gene coloning |
| *MsMTA-R* | ACATGCCATCCTCGCCTAAAT |  |
| *MsMTA-OE-F* | aggatccccgggtaccATGGACATGGAGACAGATGAAG | Overexpression vector construction |
| *MsMTA-OE-R* | gcggccgctctagaactagtTTAGTTAGTGTCCACGTCAATG |  |
| *MsMTA-RNAi-attB1F* | ggggacaagtttgtacaaaaaagcaggcttcCCCTCTCCCTCCTCCTCTAACC | RNAi vector construction |
| *MsMTA-RNAi-attB2R* | ggggaccactttgtacaagaaagctgggtcGTACTCGACTTAAAAGACAAAC |  |
| *qMsGS2-F* | CTGGCATTGGTCCTGGTGTT | m6A-IP-qPCR and mRNA stability |
| *qMsGS2-R* | GCAGGCTTAACCTCGGTGTAT |  |
| *qMsSDR1-F* | GCTGAGATCGGAACTGGTGAA |  |
| *qMsSDR1-R* | AAGGCAATAGCACGGTATCCA |  |
| *qMsLQY1-F* | ACCACTCTTCCTCCTCTTCCA |  |
| *qMsLQY1-R* | TGTGCTCGCTTCCTGTTAAGA |  |
| *qMsSCPL22-F* | GACTCCACCAAACGCAAACTC |  |
| *qMsSCPL22-R* | ACCACTGAAGACACCACAACA |  |
| *qMsLOX3 -F* | GCGGAGCGTTCAAGTTCAAG |  |
| *qMsLOX3 -R* | CGTTGCTTCTGCCACCACT |  |
| *qMsATPC2 -F* | AGGGCTTTGCAGGAATCACTT |  |
| *qMsATPC2 -R* | ATACCAAGGCATCAGCACCAG |  |
| *qMsAPT1-F* | TTGGTCCTCCCATTGCCCTA |  |
| *qMsAPT1-R* | CACACTCCACTACCTCTGCTC |  |
